# Characterization of Circulating IL-7R Positive Cell Populations for Early Detection of Pancreatic Ductal Adenocarcinoma

**DOI:** 10.3390/jcm10184157

**Published:** 2021-09-15

**Authors:** Sun-Hee Heo, Sung Ill Jang, So Young Kim, Bongkun Choi, Dong Ki Lee, Hyung Keun Lee, Eun-Ju Chang

**Affiliations:** 1 Department of Biomedical Sciences, Asan Medical Center, University of Ulsan College of Medicine, Seoul 05505, Korea; jiwoni450@hanmail.net (S.-H.H.); bkchoi89@hanmail.net (B.C.); 2 Department of Gastroenterology, Yonsei University College of Medicine, Seoul 03722, Korea; AEROJSI@yuhs.ac (S.I.J.); dklee@yuhs.ac (D.K.L.); 3 Severance Institute for Vascular and Metabolic Research, Yonsei University College of Medicine, Seoul 03722, Korea; praha91@yahoo.com; 4 Institute of Biomedical Research, Yonsei University College of Medicine, Seoul 03722, Korea; 5 Department of Ophthalmology, Institute of Vision Research, Yonsei University College of Medicine, Seoul 03722, Korea; 6 College of Pharmacy, Yonsei University, Incheon 21983, Korea; 7 Stem Cell Immunomodulation Research Center, Asan Medical Center, University of Ulsan College of Medicine, Seoul 05505, Korea; 8 Department of Biochemistry and Molecular Biology, Asan Medical Center, University of Ulsan College of Medicine, Seoul 05505, Korea

**Keywords:** interleukin-7 receptor, pancreatic cancer, memory T cell

## Abstract

(1) Background: Pancreatic cancer is a high devastating disease with the lowest survival rate among all common cancers due to difficulties in early diagnosis. The purpose of this study was to identify and characterize the distinct subset of blood cell population elevated in peripheral blood mononuclear cells (PBMC) of pancreatic cancer to evaluate the potential markers for diagnosis of pancreatic cancer; (2) Methods: We analyzed differential gene expression in PBMC from normal individuals and pancreatic cancer patients utilizing transcriptome analysis. Flow cytometry analysis was applied to identify the discrete subset of interleukin-7 receptor (IL-7R) expressing cells in these cells. The expression of IL-7R during tumorigenesis was determined in syngeneic mouse model of pancreatic cancer in vivo; (3) Results: PBMC from pancreatic cancer patients expressed elevated IL-7R mRNA compared to healthy control individuals. IL-7R expressing cells rapidly appeared from the early stages of the onset of tumor formation in syngeneic pancreatic cancer mouse model in vivo. The discrete subset of IL-7R positive cells mainly consist of naive T, central memory T, and effector memory T cells; (4) Conclusions: Taken together, our present findings suggest that pancreatic cancer patients expressed higher level of IL-7R expression in PBMC that rapidly emerged from the onset of early pancreatic tumor formation in vivo than normal individuals. Thus, it can be used as a novel biological marker for early events of pancreatic cancer development.

## 1. Introduction

Pancreatic cancer is one of the most aggressive malignancy with a five-year survival rate of as low as 10% at the time of diagnosis. This survival rate is significantly lower than the average survival rate of about 70% for all cancers. Especially in cases of distant metastasis, the five-year survival in patients with pancreatic cancer decreases to less than 3% [1,2]. The low survival rate is attributed to several factors: (1) most patient with pancreatic cancer remain asymptomatic until the disease develops to an advanced stage. (2) Even if up to 20% of patients are diagnosed with local and resectable tumors, the five-year survival rate following surgery is only up to 20% [3,4]. while approximately 80–85% of patients are diagnosed with either unresectable or metastatic disease [3,5]. (3) The major type of pancreatic cancers is pancreatic ductal adenocarcinoma (PDAC), which accounts for more than 90% of the pancreatic cancer population and is an extremely aggressive malignant tumor. In general, pancreatic cancer is complicated by early recurrence and distant metastasis, leading to resistance to chemotherapy and radiotherapy [3]. These observations have supported that the earlier stage diagnosis for pancreatic cancer is required for clinically significant improvement. Hence, characterization of the mechanisms of early immune responses, including the identification of specific cell subtypes elevated in pancreatic cancer, in association with pancreatic cancer progression is critical for more sensitive diagnostic strategies for early detection and more effective immune therapy options to improve the survival rates in the affected patients.

Interleukin 7 (IL-7) is a cytokine produced predominantly by stromal cells in the bone marrow, thymus, and a range of other organs [6]. Its heterodimeric receptor (IL-7R), comprised of comprised of the IL-7Rα (CD127) and the common gamma chain (γc; CD132), which is shared by the receptors for IL-2, IL-4, IL-9, IL-15 and IL-21, is mainly expressed in lymphoid lineage cells [6]. This IL7/IL-7R signaling machinery is required for normal and memory T-cell development as well as homeostasis of mature T-cells under normal physiological conditions [7]. Accordingly, dysregulation of the IL-7/IL-7R axis has been implicated in the pathogenesis of different disease states including diabetes, multiple sclerosis, and rheumatoid arthritis due to dysregulation of lymphoid function [8,9,10]. Moreover, the crucial role of IL-7/IL-7R axis is especially relevant in the context of cancer. Indeed, IL-7/IL-7R-mediated signaling plays an oncogenic role in hematological tumors by may being resistant to conventional chemotherapy and targeted therapeutics [11,12]. Beyond hematological cancer, high expression of IL-7 and IL-7R in tumor tissues of breast and lung cancer patients is positively correlated with more aggressive breast cancers and poor survival [13,14,15,16]. Lung cancer patients carrying IL-7R rs10213865 (AGAA haplotype) have higher lung adenocarcinoma risk [17]. However, there is few data for elucidation of IL-7R level in blood cells and tumor cells from tumor patients. Moreover, little was known about its involvement in pancreatic cancer.

The aim of this study was to identify and characterize the distinct subset of blood cells elevated in pancreatic cancer. Differentially expressed mRNA in PBMC was analyzed in human healthy subjects and pancreatic cancer patients to reveal the positive correlation between IL-7R expression in PBMC and pancreatic cancer utilizing transcriptome analysis. We found that protein expression of IL-7R was rapidly increased from the early stages of the onset of tumor formation in orthotopic pancreatic cancer mouse model in vivo and identified the discrete subset of IL-7R expressing cells. Our results suggest that IL-7R could be a potential marker for early pancreatic cancer diagnosis.

## 2. Materials and Methods

### 2.1. Patients and Samples

Blood samples were obtained from 7 healthy individuals and 15 pancreatic cancer patients for the identification of pancreatic cancer-selective genes by transcriptome analysis. For analysis of correlation IL-7R expression using flow cytometry, peripheral blood samples were obtained from normal individuals and patients with pancreatic cancer (*n* = 7). To analyze IL-7 protein expression, blood samples collected from pancreatic cancer patients were separated into PBMC and serum. For characterization of IL-7Rα expressing cells in T lymphocytes with several disease types using flow cytometry, peripheral blood samples were obtained from patients with pancreatic cancer (*n* = 6) and other disease (*n* = 8) including acute pancreatitis (*n* = 1), pancreatic cysts (*n* = 1), chronic pancreatitis (*n* = 1), bile duct cancer (*n* = 3), lung cancer (*n* = 1), and cholangiocellular carcinoma (*n* = 1). This study was approved by the institutional review board (IRB) of Gangnam Severance Hospital (IRB No. 3-2018-0293, 3-2015-0012) and registered with the clinical research information service (CRIS, https://cris.nih.go.kr/cris/en/, KCT0004614, accessed on 8 January 2020). Informed consent was obtained from all donors and this study was performed in accordance with the Declaration of Helsinki. The study was performed from January 2020 to April 2021.

### 2.2. Pancreatic Cancer Mouse Model

The animals were maintained in a specific pathogen-free environment ad were acclimated for at least 1 week before any experiments were performed. All procedures were approved by the Animal Care and Use Committee at Yonsei University College of Medicine and conducted in accordance with the Guide for the Care and Use of Laboratory Animals (National Institutes of Health, no. 85-23, 1996), and the ARRIVE guidelines. Eight- to 10-week-old C57BL/6 mice were randomly assigned and the mouse pancreatic cancer Pan02 cells were orthotopically implanted into the mouse pancreas. The mice were sacrificed on a scheduled date. Tumor and spleen from each mouse were excised and weighted. PBMC was separated and used for flow cytometry analysis of IL-7R expression using mouse IL-7R specific antibody.

### 2.3. IL-7R Expression Analysis

#### 2.3.1. mRNA-Seq Data

Transcriptome analysis was performed using total RNA extracted from PBMC of healthy donors (*n* = 7) and pancreatic cancer patients (*n* = 15). The seven normal donors were average 65 years old and 15 individuals with pancreatic cancer were 67.2 years old.

Total RNA was used to construct cDNA libraries with the TruSeq Stranded mRNA LT Sample Prep Kit. The protocol consisted of polyA-selected RNA extraction, RNA fragmentation, random hexamer primed reverse transcription and 100 nt paired-end sequencing by Illumina NovaSeq 6000. The libraries were quantified using qPCR according to the qPCR Quantification Protocol Guide and qualified using an Agilent Technologies 2100 Bioanalyzer (Agilent. Technologies, Inc., Palo Alto, CA, USA).

We preprocessed the raw reads from the sequencer to remove low quality and adapter sequences before analysis and aligned the processed reads to Homo sapiens (hg19) using HISAT v2.1.0 [18]. HISAT utilizes two types of indexes for alignment (a global, whole-genome index and tens of thousands of small local indexes). These two index types were constructed using the same Burrows–Wheeler transform (BWT) and graph FM index (GFM) as Bowtie2. Because it uses efficient data structures and algorithms, HISAT generates spliced alignments several times faster than Bowtie and BWA, two widely used methods. The reference genome sequence of Homo sapiens (hg19) and annotation data were downloaded from NCBI and the transcript assembly of known transcripts was processed by StringTie v1.3.4d [19,20]. Then, the expression abundance of transcripts and genes was calculated as read counts or Fragments Per Kilobase of exon per Million fragments mapped (FPKM) values per sample. The expression profiles were used for additional analysis such as Differentially Expressed Genes (DEG). In groups with different conditions, DEG or transcripts can be filtered through statistical hypothesis testing.

#### 2.3.2. Statistical Analysis of Gene Expression Levels

The relative abundances of genes were measured in read counts using StringTie (https://ccb.jhu.edu/software/stringtie/. Version 2.1.3b updated on and accessed on 5 December 2020). We performed the statistical analysis to find differentially expressed genes using the estimates of abundances for each gene in samples. Genes with one more than zeroed read count value in the samples were excluded. Filtered data were log2-transformed and subjected to RLE Normalization. Statistical significance of the differential expression data was determined using DESeq2 nbinomWaldTest [21] and fold change in which the null hypothesis was that no difference exists among groups. The false discovery rate (FDR) was controlled by adjusting the *p* value using the Benjamini-Hochberg algorithm. After normalization and filtering, 257 genes were selected as the DEG set. For the DEG set, hierarchical clustering analysis was performed using complete linkage and Euclidean distance as a measure of similarity. Enrichment of gene ontology analysis was performed for DEGs using g:Profiler [22] and KEGG pathway analysis was tested using the KEGG pathway database (https://www.genome.jp/kegg/, version updated on and accessed on 6 July 2020).

#### 2.3.3. Multidimensional Scaling

We used a multidimensional scaling (MDS) method to visualize the similarities among samples. MDS converts the structure in the similarity matrix to a simple geometrical picture as a scatter plot. The larger the dissimilarity between 2 samples, the further apart the points representing the experiments in the picture are. We applied this to the Euclidean distance as a measure of the dissimilarity.

#### 2.3.4. Hierarchical Clustering

Hierarchical clustering analysis was also performed using complete linkage and Euclidean distance as a measure of similarity to determine the expression patterns of differentially expressed transcripts that were satisfied by |fold change| ≥ 2 and raw *p* < 0.05.

All data analysis and visualization of DEG was conducted using R 3.6.3 (www.r-project.org, accessed on 29 February 2020).

#### 2.3.5. RNA HPA Tissue Gene Data

The pathology atlas of the human cancer transcriptome, part of the Human Protein Atlas (http://v20.proteinatlas.org; www.proteinatlas.org/pathology, Version 20.0 updated on and accessed on 19 November 2020) offers summarized transcript expression levels per gene in 37 tissues based on RNA-seq [23]. The expression distribution of IL-7R in pancreatic cancer patient samples was evaluated and the survival probability of pancreatic cancer patients was analyzed according to IL-7R expression based on a cut-off value of 2.12.

### 2.4. Flow Cytometry

The following antibodies were used for flow cytometry analysis: IL-7R-PE, CD45RA-APC, CD3-APC/Cy7, CD4-Alexa Fluor 488, CD8-PE/Cy7, CD14-PerCP/Cy5.5, CD16-Alexa Fluor 488, CD95-PerCP/Cy5.5, CCR7-PE/Cy7, HLA-DR-APC, and isotype controls (mouse IgG1K Isotype Control Alexa Fluor 488, mouse IgG1 K Isotype Control PE, mouse IgG1 K Isotype Control PE/Cy7, mouse IgG2a K Isotype Control PE/Cy7, mouse IgG1 K Isotype Control PerCP/Cy5.5, mouse IgG2a K Isotype Control APC, mouse IgG2b K Isotype Control APC, mouse IgG2a K Isotype Control APC/Cy7) were purchased from BioLegend (San Diego, CA, USA).

For IL-7R expressing population analysis, single cells suspended in 500 mL phosphate buffered saline (PBS) with containing 2% fetal bovine serum (FBS) and incubated with fluorescent conjugated antibodies for 1 hour at 4 °C. The labeled cells washed with PBS and resuspended with 200 mL 0.5% FBS. Multiparameter analysis was performed on FACSCanto™ II Cell Analyzer (BD Biosciences, San Jose, CA, USA) and the data were analyzed with FlowJo software. All flow cytometric analysis of immune cells was performed on live DAPI negative region. Initial gating was executed for mononuclear populations using FSC-A/SSC-A profiles. After the doublets and dead cells were excluded using FSC-A/FSC-H profiles and SSC-A/DAPI profiles, respectively. To optimize compensation, the single-stained samples and Fluorescence-Minus-One controls were used. Subsequent gating was designed for characterization of IL-17Ra expressing populations in PBMCs or for composition analysis of IL-7Ra+ memory subsets in T cells.

### 2.5. Statistical Analyses

All statistical analyses were carried out using SPSS Version 25 (SPSS, Chicago, IL, USA) and GraphPad Prism 5 (GraphPad Software Inc., La Jolla, CA, USA). All tests were two-sided and were performed with a significance level of *p* < 0.05, *p* < 0.01 and *p* < 0.001. 

## 3. Results

### 3.1. Analysis for Pancreatic Cancer-Specific Genes

**Figure 1:**
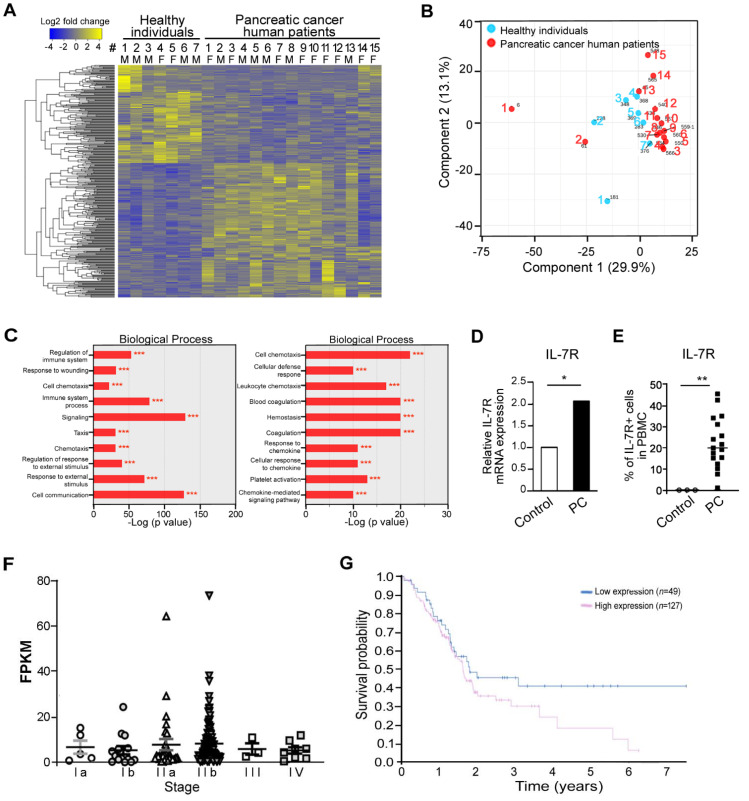
Transcriptomic analysis of PBMCs from normal individuals and pancreatic cancer patients for biomarker discovery. (**A**) Heatmap showing relative gene expression [log2FPKM (fragments per kilobase million)] of 364 differentially expressed genes (DEGs) in PBMC between 7 normal healthy donors and 15 pancreatic cancer patients. Yellow and blue reflect high and low expression levels, respectively, as indicated in the scale bar (log 2 transformed scale). Each row represents a DEG, and each column represents a sample. List of differentially expressed genes are presented. (**B**) Multidimensional scaling of transcriptome analyses in these cells. Principal component analysis of transcriptome of healthy individuals (blue) and pancreatic cancer human patients (red), using gene expression counts for each group. Each dot represents one sample. (**C**) Top 20 biological processes associated with genes differentially expressed in PBMCs from pancreatic cancer patients. *** *p* < 0.001 versus healthy controls. (**D**) Elevated mRNA expression of IL-7R in PBMCs from pancreatic cancer (PC) patients as revealed by transcriptomic analysis. * *p* < 0.05 versus control. (**E**) Increased IL-7R positive cells in PBMCs from pancreatic cancer (PC) patients compared with healthy controls (Control). ** *p* < 0.01 versus control. (**F**) Analysis of IL-7R expression in pancreatic cancer of the human protein atlas. (**G**) Kaplan-Meier survival curves for PDAC patients with low IL-7R expression or high IL-7R expression (http://v20.proteinatlas.org; www.proteinatlas.org/pathology, Version 20.0 updated on and accessed on 19 November 2020).

To identify differentially expressed genes in PBMCs from pancreatic cancer patient, Transcriptome analysis was performed using PBMCs of 7 normal healthy donors and 15 pancreatic cancer patients. As described in Material and Methods, the transcripts were filtered by their fold changes (>2) and *p*-values (<0.05) based on FPKM value in comparison analysis. By using this critical, 364 differentially expressed genes with *p* values < 0.05 in PBMCs from pancreatic cancer patients are represented (Figure 1A, blue/yellow heatmap). In addition, statistical significance was analyzed based on sex but there was no statistical significance in normal healthy donors (*p* > 0.9586) or pancreatic cancer patients (*p* > 0.5365) between female and male group. The majority of pancreatic cancer patients (13/15) tended to separate from healthy control group along the principal component 1 (Figure 1B). Gene ontology enrichment analysis of differentially expressed genes showed “Immune system process”, “Signaling”, “Response to external stimulus”, and “Cell communication” as the top 4 enriched biological processes (Figure 1C). Notably, IL-7R expression was significantly up-regulated in PBMCs from pancreatic cancer patients in comparison of those of healthy donors (Figure 1D), indicating that IL-7R mRNA expression is elevated in PBMC from patients with pancreatic cancer.

IL-7R positive cells were measured in PBMCs from pancreatic cancer patients and healthy donors. IL-7R positive cells constituted 0.01% (range, 0.008–0.020) of normal PBMCs and 22.1% (range, 1.100–45.200) of PBMCs from pancreatic cancer patients. IL-7R positive cells were significantly increased in PBMCs from pancreatic cancer patients compared with PBMCs from healthy donors (Figure 1E).

In addition, the expression of IL-7R mRNA in pancreatic cancer patients with various stages was also evaluated utilizing pathology atlas of the human cancer transcriptome obtained from open-access interactive database (Human Protein Atlas, the Version 20.0 updated on and accessed on 19 November 2020). The IL-7R expression levels in the early stages including Ia, Ib, IIA and IIB appears to be higher than those in the relatively later stages, staged III and IV, however, the difference was not statistically significant (Figure 1F). Moreover, Kaplan-Meier curves showed that survival probability for PDAC patients with low IL-7R expression is modestly higher than that for patients with high IL-7R expression (*p* = 0.14, Figure 1G).

### 3.2. Elevated Expression of IL-7R during Tumorigenesis in Pancreatic Cancer Murine Model In Vivo 

**Figure 2:**
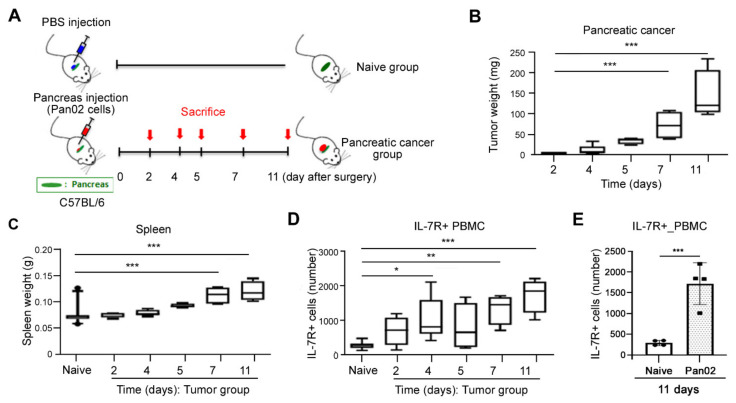
Elevation of IL-7Ra expressing cells during early tumorigenesis in vivo. (**A**) Schematic illustration of an orthotopic syngeneic pancreatic cancer murine model. Pan02 cells were transplanted into pancreas. Four mouse bearing pancreatic cancer per group were sacrificed after 2, 4, 5, 7, 11 days. Mice injected with PBS was used as a naive control (N = 15). (**B**,**C**) On the day of sacrifice, tumors (**B**) and spleens (**C**) were removed and weighted in syngeneic mouse model. *** *p* < 0.001 versus day 2 (**B**) or naive control (**C**). (**D**) The number of IL-7R expressing cells were determined from mouse PBMC during tumorigenesis in vivo. * *p* < 0.05, ** *p* < 0.005, or *** *p* < 0.001 versus naive control. (**E**) IL-7R expression in PBMC was analyzed from naive or tumor bearing mice after 11 days of Pan02 injection.

We showed that IL-7R expression in pancreatic cancer patients was elevated compared to healthy human subjects (Figure 1). To confirm the positive correlation between IL-7R expression in PBMC and the tumorigenesis of pancreatic cancer, we applied syngeneic mouse model of PDAC. Mouse PDAC Pan02 cells were transplanted into pancreas and sacrificed after 2, 4, 5, 7, 11 days of Pan02 injection (Figure 2A). On the day of sacrifice, tumors and spleens were removed and weighted. Tumors were formed in all mice even on 2 days after injection of pancreatic cancer cells, and the average weight of the formed tumor tissue was 3.42 mg (range, 1.5–4.4 mg, Figure 2B). The tumor weight was gradually increased thereafter and significantly increased after 7 and 11 days of Pan02 injection (Figure 2B). The spleen weight was also increased after 7 and 11 days of pancreatic cancer cell injection (Figure 2C). Importantly, the number of IL-7R positive (IL-7R+) cells in PBMC began to be detected even on 2 days after Pan02 injection and its expression was gradually upregulated until the end of the experiment at 11 days (Figure 2D,E). It was confirmed that IL-7R+ cells in PBMC were higher in tumor cell bearing mouse than PBS injected mouse (Figure 2E). This rapid appearance of IL-7R+ cells during tumorigenesis of pancreatic tumor indicates that IL-7R may be potentially useful as an early diagnosis marker for pancreatic cancer development.

### 3.3. Characterization of Discrete Subset of IL-7R Expressing Cells

**Figure 3:**
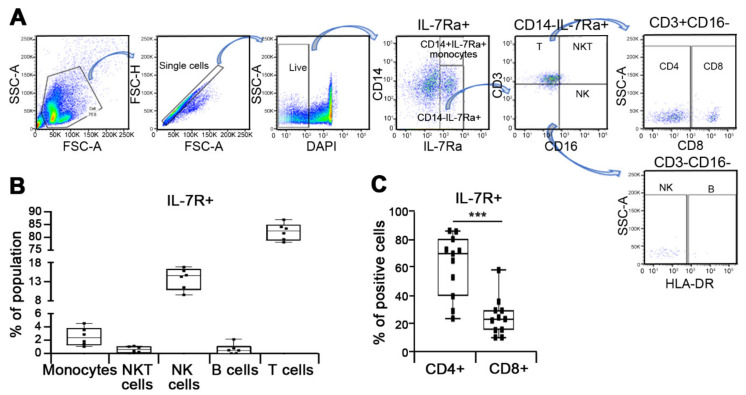
Prominent expression of IL-7Ra in T lymphocytes. (**A**) Representative gating strategy for identification of IL-7Ra expressing cells based on expression of immune cell markers including CD3, CD8, CD14, CD16 and HLA-DR to define monocytes, natural killer T (NKT) cells, natural killer (NK) cells, B lymphocytes, and T lymphocytes in PBMCs. (**B**) PBMC were isolated from pancreatic cancer patients (*n* = 6). The percentage of IL-7R positive (IL-7R+) cells was determined utilizing flow cytometry analysis in monocytes, NK cells, NKT cells, T lymphocytes, and B lymphocytes. (**C**) The percentage of IL-7R+ cells among CD4+ or CD8+ cells from pancreatic cancer patients (*n* = 11) was evaluated by flow cytometry analysis. *** *p* < 0.001 versus CD4+ (mean ± SD).

Given the elevated expression of IL-7R in PBMC from pancreatic cancer patients (Figure 1) and rapid appearance of IL-7R+ cells during tumorigenesis in murine model (Figure 2), we next performed flow cytometry analysis using several markers of immune cells in order to identify the discrete subpopulation expressing IL-7R in PMBC from pancreatic cancer patients. PBMCs were isolated from 7 pancreatic cancer patients. CD3, CD4, CD8, CD14, CD16 and HLA-DR markers were applied to distinguish monocytes (CD14+), natural killer T cells (CD14-CD16+CD3+), T lymphocytes (CD14-CD16-CD3+), B lymphocytes (CD16-CD3-HLA-DR+), and natural killer cells (CD14-CD16+CD3- or CD16-CD3-HLA-DR-) (Figure 3A). We observed that the discrete subset of IL-7R expressing cells mainly consists of CD3 positive (CD3+) T cells (Figure 3B). Among CD3+ T cells, the percentage of IL-7R+ cells among CD4+ cells was relatively higher than that of CD8+ cells (Figure 3C), suggesting that IL-7Ra is predominantly expressed in CD4+CD3+ T lymphocytes.

**Figure 4:**
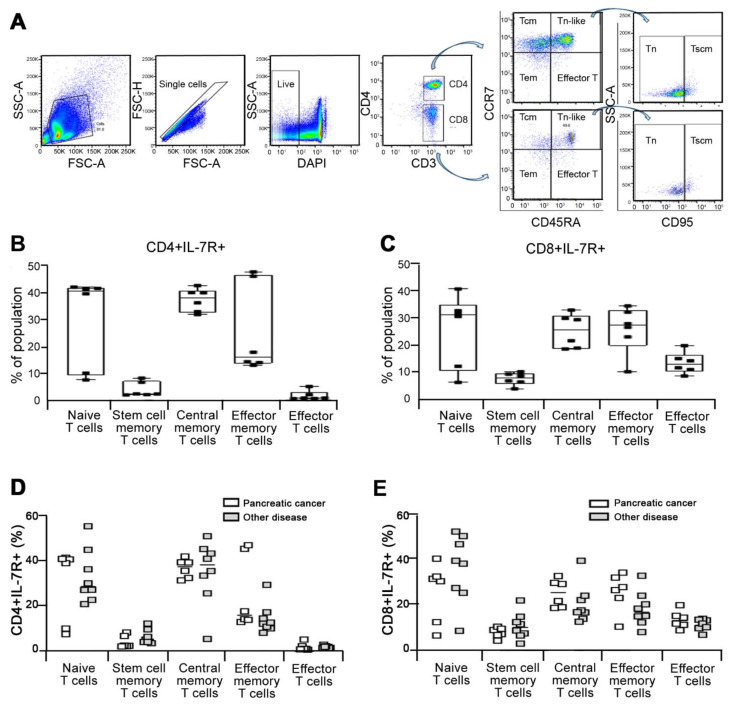
Characterization of IL-7Rα expressing cells in T lymphocytes. (**A**) Representative gating strategy for identification of IL-7Ra expressing cells in T lymphocytes based on expression of cell markers including CD3, CD4, CD45RA, CCR7, and CD95 to define naive (CD45RA+CCR7+CD95-), stem cell-like memory (Tscm, CD45RA+CCR7+CD95+), central memory (Tcm, CD45RA-CCR7+), effector memory (Tem, CD45RA-CCR7-) and effector T cells (CD45RA+CCR7-) among CD4+CD3+ T cells. (**B**,**C**) PBMC were isolated from pancreatic cancer patients (*n* = 6). The abundance of IL-7R+ cells were evaluated in naive, stem cell-like memory, central memory, effector memory, and effector T cells among CD4+CD3+ T (**B**) and CD8+CD3+ T (**C**) cells in PBMC isolated from patients with pancreatic cancer (*n* = 6) and other disease (*n* = 8) including acute pancreatitis (*n* = 1), pancreatic cysts (*n* = 1), chronic pancreatitis (*n* = 1), bile duct cancer (*n* = 3), lung cancer (*n* = 1), and cholangiocellular carcinoma (*n* = 1) by flow cytometry analysis. (**D**,**E**) The percentage of IL-7R+ cells was determined in these cells among CD4+CD3+ T (**D**) and CD8+CD3+ T (**E**) cells from (**B**,**C**).

We showed that IL-7Ra is predominantly expressed in T lymphocytes and thus further dissect the subset of IL-7R+ cells in CD3+ T cells. CD4+CD3+IL-7R+ or CD8+CD3+IL-7R+ cells are classified into naive (CD45RA+CCR7+CD95-), stem cell-like memory (Tscm, CD45RA+CCR7+CD95+), central memory (Tcm, CD45RA-CCR7+), effector memory (Tem, CD45RA-CCR7-) and effector T cells (CD45RA+CCR7-) depending on expression of CD45RA, CCR7 and CD95 [24,25] (Figure 4A). Naïve T, central memory T, and effector memory T cells relatively expressed higher level of IL-7R than those of stem cell memory T and effector T cells in both CD4+ (Figure 4B) and CD8+ cells (Figure 4C). We then compared the percentage of IL-7R+ in these cells between pancreatic cancer patients (*n* = 6) and other disease patients (*n* = 8). The percentage of effector memory T population in CD4+CD3+IL-17R+ (Figure 4D) and CD8+CD3+IL-17R+ (Figure 4E) population appears to be higher in pancreatic cancer patients compared to other disease group whilst the percentage of naive T cells in pancreatic cancer patients tends to be decreased in both CD4+CD3+IL-17R+ (Figure 4D) and CD8+CD3+IL-17R+ (Figure 4E) cells. These results suggest that the discrete subset of IL-7R+ cells mainly consist of naive T, central memory T, and effector memory T cells in T lymphocytes.

## 4. Discussion

IL-7 is known to induce the differentiation and development of haematological malignancies, such as lymphomas [26,27] and leukaemias [28]. Aberrantly elevated expression of IL-7 and its signaling complex including Jak-3 and PI3-K is associated with aggressive human breast cancer [13] and IL-7 significantly accelerates the growth of breast cancer cells in vitro [15]. Moreover, IL-7/IL-7R-induced VEGF-D upregulation positively correlates with lymph node metastasis, clinical stages, and survival in human non-small cell lung cancer patients [16]. Elevated IL-7 in prostate cancer patients with early stage also promotes the invasiveness of prostate cancer cells by inducing epithelial–mesenchymal transition [26,29]. These several lines of evidence suggest that aberrantly elevated IL-7R may promote the differentiation and development of a variety of tumor, however, there is no study relating the level of IL-7R expression to the histopathological characteristics of pancreatic cancer to date. Moreover, in the above studies measured IL-7R levels in tumor tissue but not the immune cells, tumor IL-7R expression levels in tumor tissue is hardly usable for detection of early cancer.

Pancreatic cancer is one of highly aggressive malignancies with a poor survival rate, largely due to the lack of available diagnostic biomarkers for early detection. Thus, biomarkers being able to detect pancreatic cancer at the early stage can benefit the effective screen for high-risk pancreatic cancer patients [30,31], leading to the increased survival rates by providing appropriate treatment options. We here showed that IL-7R+ cells in PBMCs rapidly appeared from the early stages of the onset of tumor formation in syngeneic pancreatic cancer mouse model in vivo (Figure 2) and the expression of IL-7R was significantly elevated in pancreatic cancer patients compared to healthy individuals (Figure 1). However, since these results are preliminary, further studies are necessary to verify IL-7R being an effective prognostic marker with large-scale validation containing healthy subjects. Furthermore, further studies should consider the biological factors of the disease, such as sex. In addition, the gene expression related to immune response was mainly altered in PBMC form pancreatic cancer patient compared to normal donors, which may imply aggressive immune reaction in disease condition (Figure 1C). These results indicate that IL-7R may be useful as a potential biomarker for early detection of pancreatic cancer. Indeed, we have analyzed the ability of differential diagnostic power with levels of IL-7R among healthy subjects, pancreatic cancer, and other pancreatic diseases (e.g., chronic pancreatitis) for a large-scale validation and found the higher differential levels between the diseases.

In the current study, we showed that pancreatic cancer patients exhibited increased number of effector memory type cells (CCR7-CD45RA-) and decreased number of naive T cells compared to other disease group (Figure 4). In similar to tissue-resident CD103+IL-7R+ Tregs, these amplified effector memory T cells population (CCR7-CD45RA-CD3+IL-7R+) may contribute to constitute immunosuppressive environment in early onset of tumor formation [32,33]. Moreover, IL-7R expressing effector memory T cells derived from naive T cells possess high affinity to IL-2 and thereby enhance the survival of themselves in tumor microenvironment [34].

In conclusion, we showed that IL-7R expressing cells in PBMC was detectable in early period during tumor formation in vivo. Moreover, IL-7R in PBMC was highly expressed in patients with pancreatic cancer compared to healthy donors. We also confirmed that the IL-7R expressing cells mainly consists of CD3+ T cells, especially CD3+CD4+ T cells. These results suggest that elevated IL-7R expression in PBMC may be useful as an early diagnosis marker for pancreatic cancer.

## Data Availability

The data presented in this study are available on request from the corresponding author.
